# Defecation Site Preferences and Spatial Ecological Segregation of Forest Musk Deer and Siberian Roe Deer in North China

**DOI:** 10.3390/ani15010061

**Published:** 2024-12-30

**Authors:** Yixin Li, Luyao Hai, Pengfei Luo, Wangshan Zheng, Xuelin Jin, Jiangcheng Liu, Haiyan Wang, Defu Hu

**Affiliations:** 1 School of Ecology and Nature Conservation, Beijing Forestry University, Beijing 100107, China; lyx010805@bjfu.edu.cn (Y.L.);; 2 Shaanxi Institute of Zoology, Xi’an 710032, China; 3 Yanan Huanglongshan Forestry Bureau, Yan’an 715700, China; 4 School of Information Science and Technology, Beijing Forestry University, Beijing 100107, China

**Keywords:** forest musk deer, Siberian roe dee, defecation site, microhabitat selection, generalized linear mixed model

## Abstract

The forest musk deer (*Moschus berezovskii*) and Siberian roe deer (*Capreolus pygargus*) are browsers with a broad sympatric distribution in North and Southwest China. However, little is known about their spatial utilization of microhabitats and habitats. This study, conducted on Huanglong Mountain in China, analyzed the defecation site distribution, indicating preferences of forest musk deer and Siberian roe deer for their habitat demands. Using generalized linear mixed models (GLMMs), we compared the defecation site preferences of both species and further examined their spatial utilization patterns. The results indicated that the primary factors influencing defecation site preferences for forest musk deer were slope (15.79%), elevation (4.26%), herbaceous cover (19.93%), herb height (33.73%), and tree diversity (15.64%). Conversely, for Siberian roe deer, elevation (54.63%) and herbaceous cover (29.31%) were the key factors. Significant differences were found in elevation (*p* < 0.001) and herbaceous diversity (*p* < 0.01) between the defecation sites of the two species, with additional notable differences in slope position, tree diversity, and average tree height (*p* < 0.05). Furthermore, forest musk deer primarily utilized broadleaf forests, coniferous forests, mixed conifer-broadleaf forests, and sparse woodlands. In contrast, Siberian roe deer utilized broadleaf forests, sparse woodlands, and coniferous forests, showing a significant difference (*p* = 0.01). These findings suggest distinct spatial ecological segregation between forest musk deer and Siberian roe deer regarding their microhabitat preferences and vegetation type utilization at the habitat scale.

## Simple Summary

Studying the distribution of defecation sites is a common technique for determining the preferred habitats of different animal species. We compared the defecation site preferences and spatial ecological segregation between two sympatric species, the forest musk deer (*Moschus berezovskii*) and Siberian roe deer (*Capreolus pygargus*), on Huanglong Mountain, North China. Overall, forest musk deer exhibited a more specialized defecation site selection compared to Siberian roe deer, with significant spatial ecological segregation observed between the two species. The results of this study provide valuable insights for the development and implementation of more targeted conservation strategies and management measures in the local area.

## 1. Introduction

Species coexistence refers to the phenomenon where two or more species occupy the same habitat and maintain stable population levels through resource partitioning [1]. When species use the same limited resources within the same geographical space, interspecific competition occurs. Such competition is likely to affect how each species utilizes these resources. Therefore, comparative studies on resource utilization among sympatric species are essential for understanding interspecific interactions and their operational mechanisms [2]. In fact, sympatric species often exhibit clear resource partitioning, which may have evolved due to past competition [3]. Large herbivores play a crucial role in shaping the composition and structure of forest ecosystems, and interspecific competition may have significant impacts on ungulate populations within these systems [4]. Current research on the coexistence of ungulate species primarily focuses on the degree of separation in their spatiotemporal niches and trophic dimensions [5,6,7].

In the Huanglong Mountain region of Shaanxi, forest musk deer (*Moschus berezovskii*) and Siberian roe deer (*Capreolus pygargus*) are sympatric dominant ungulate species. The forest musk deer, a forest-dwelling species endemic to East Asia, is primarily distributed in China and northern Vietnam [8,9,10]. In contrast, the Siberian roe deer is a small ungulate species widely distributed across northern Eurasian forests [11,12]. Both species exhibit extensive sympatric distribution in the zoogeographical North China and Southwest China regions [13,14]. Both species are key prey for medium to large carnivores in forest ecosystems, playing important ecological roles. In recent decades, due to deforestation, the planting of monoculture plantations, and prolonged illegal hunting, the populations of wild forest musk deer and roe deer have declined sharply, and their distribution range has progressively contracted [15].

Defecation sites serve as critical chemical communication venues where wildlife can release pheromones through excreta [16] for social interactions. For species exhibiting latrine behavior, such as forest musk deer, fixed defecation sites also function as territorial markers, providing signals to conspecifics [17,18,19,20,21]. Moreover, defecation sites serve as important indicators of microhabitat selection in wildlife [18,22,23,24]. These sites are often considered relatively safe, where animals are relaxed, providing a basis for studying their microhabitat choices [18,25,26,27]. In areas that contain two or more species of the same foraging group, defecation sites reflect spatial utilization among different species at the microhabitat scale [3,28]. Both forest musk deer and Siberian roe deer are classified as concentrate selectors within the same trophic level [29]. Investigating their preferences for defecation sites and ecological separation can deepen our scientific understanding of their coexistence strategies, facilitating the development of effective conservation planning and habitat management.

Current spatial studies of the forest musk deer and Siberian roe deer primarily focus on distribution [30,31], home range [32,33,34], habitat selection [35,36,37,38,39,40,41,42], and preferences for defecation sites [18,25,27,43,44,45]. However, no research has been reported on the defecation sites and microhabitat preferences of sympatric forest musk deer and Siberian roe deer. Addressing this knowledge gap is crucial for understanding the coexistence of these two species. Furthermore, it provides a solid scientific foundation for developing effective conservation strategies.

This study conducts a comparative analysis of the defecation site habitats of forest musk deer and Siberian roe deer in the Huanglong Mountain region of Shaanxi Province. It identifies the key environmental factors influencing defecation site selection for both species and explains their different preferences, with the aim of providing scientific support for the population recovery and conservation management of these two species.

## 2. Materials and Methods

### 2.1. Study Area

**Figure 1:**
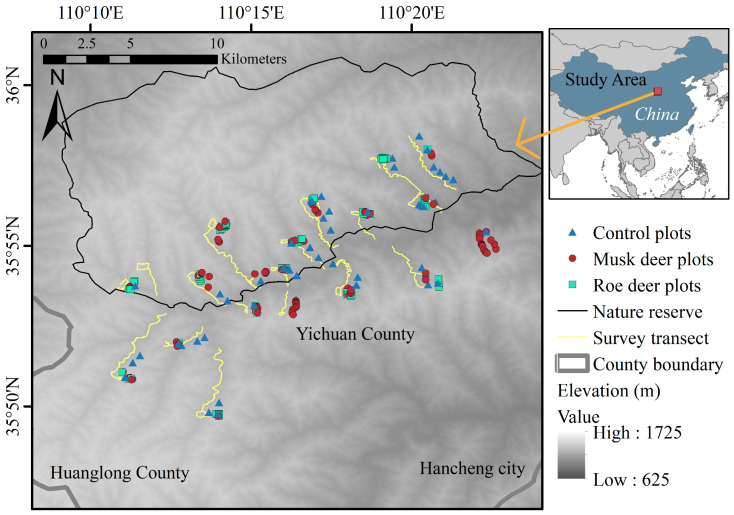
Geographic location of the study area and placement of sampling plots and transects.

The Huanglong region is located in central-eastern Shaanxi Province, China, serving as a transitional zone from the hilly and gully areas of the Loess Plateau to the Guanzhong Plain. This study area is situated within the provincial-level natural reserve for forest musk deer in Yichuan County, Yan’an City, Shaanxi Province (110°07′–110°31′ E, 35°47′–36°00′ N). This region features continuous mountain ranges and numerous gullies, with elevations ranging from 625 m to 1725 m (Figure 1). It experiences a warm, temperate, semi-arid climate, with an average annual temperature of 10.25 °C and an average yearly precipitation of 549.3 mm [46]. The topography includes narrow valleys, relatively steep mid-to-lower mountain slopes, and gentle upper slopes. Vegetation types include sparse forest shrubs, broadleaf forests, mixed coniferous and broadleaf forests, and coniferous forests. The mountains have a slight elevation difference. Additionally, the vertical vegetation characteristics are not prominent, creating a mosaic distribution. Key tree and shrub species include Liaodong oak (*Quercus liaotungensis*), oil pine (*Pinus tabuliformis*), sea buckthorn (*Hippophae rhamnoides*), white birch (*Betula platyphylla*), smoke tree (*Cotinus coggygria*), and mountain peach (*Amygdalus davidiana*) [47]. The main wildlife species protected in this reserve include the leopard (*Panthera pardus*), leopard cat (*Prionailurus bengalensis*), forest musk deer (*Moschus berezovskii*), Siberian roe deer (*Capreolus pygargus*), and brown-eared pheasant (*Crossoptilon mantchuricum*) [48].

### 2.2. Field Survey and Data Collection

The survey for this study was conducted from May to June 2024. We first reviewed infrared camera data deployed within the protected area. These cameras recorded locations where forest musk deer and Siberian roe deer had been previously captured. This was followed by consultations with experienced forest rangers to identify regions where forest musk deer and Siberian roe deer may be present. Guided by the rangers, we established 19 sampling transects, each at least 2 km long. While recording the locations, we searched for and collected feces from ungulates located within 5 m on either side of each transect.

A 10 × 10 m tree plot and a 5 × 5 m shrub plot were established upon finding feces. Four 1 × 1 m herbaceous plots were also set up at the corners of each tree plot. Random control plots were also established 500 m from the defecation sites to reflect the overall habitat characteristics [25]. Based on relevant literature regarding forest musk deer and roe deer, 18 key predictor variables potentially influencing defecation site preferences were recorded (Table 1). Among these predictors, the five categories of slope position—valley bottom, lower slope, mid-slope, upper slope, and ridge—were defined as numerical variables ranging from 1 to 5. To avoid circularity issues regarding the slope aspect, this was converted into a continuous variable between 0 and 1 [49] using the following formula:

Asp = [1 − cos((π/180) × (α − 30))]/2,(1)

where α represents the slope aspect. This transformation assigns a value of 0 to northeast-facing slopes (typically the coolest and most humid direction) and a value of 1 to southwest-facing slopes, which are generally hotter and drier.

### 2.3. Species Identification

During field surveys, ungulate fecal pellets were collected using disposable polyethylene gloves, focusing on 5 to 15 mm long pellets. These samples were placed in 50 mL sterile tubes pre-filled with silica gel desiccant and stored at −20 °C. Species identification was conducted using fecal DNA methods, following protocols from Piggott and Taylor [50] and Tang [51] for extracting fecal DNA. We also utilized the Blood/Cell/Tissue Genomic DNA Extraction Kit (DP304) from Tiangen Biotech (Beijing) Co., Ltd. (Beijing, China).

For molecular identification, the mtDNA D-loop region was selected. Standard primers were synthesized by Shanghai Sangon Biological Engineering Technology & Services Co., Ltd. (Shanghai, China). PCR amplification was performed using DNA extracted from the fecal samples, with primer sequences and reaction conditions detailed by Feng [52] and Wang [53]. Following PCR amplification, products were evaluated via electrophoresis. Clear and single bands of the expected length were sent to Shanghai Sangon Biological Engineering Technology & Services Co., Ltd. for bidirectional Sanger sequencing. Furthermore, sequences were identified using BLAST online comparison.

If the feces in a sample plot originated from forest musk deer, it was designated as a forest musk deer plot; if they were from roe deer, it was selected as a roe deer plot. If feces in a sample plot originated from both species, it was classified as a coexistence plot; other plots were treated as control plots. In analyzing the defecation site preferences of forest musk deer, forest musk deer plots and coexistence plots were considered utilized plots. In contrast, empty plots and roe deer plots served as controls. Conversely, forest musk deer plots and coexistence plots served as control plots for analyzing the defecation site preferences of roe deer.

### 2.4. Data Analysis

We developed defecation site preference models for forest musk deer (*Moschus berezovskii*) and Siberian roe deer (*Capreolus pygargus*) using multivariate logistic regression based on generalized linear mixed models (GLMMs) in R x 64 4.2.3 [54]. GLMMs provide more accurate assessments, particularly for nested sampling, repeated measures with unbalanced designs, and modeling spatiotemporal autocorrelation structures [55]. To reduce biases caused by high multicollinearity among variables, we first conducted a multicollinearity test for the selected independent variables using the variance inflation factor (VIF) function in the “Faraway” package version 1.0.8 [56]. Variables were retained if they met the criteria of a tolerance value greater than 0.1 and a VIF value below 5.

After identifying suitable variables, we constructed GLMMs using the “lme4” package version 1.1-35.1 [57]. Because the 19 transects in the study area were spaced at certain intervals, habitat information between transects might differ. Furthermore, transects in close proximity might introduce correlations between quadrats along the same transect. To account for this, we incorporated the 19 transects as random effects and the quadrat data as fixed effects in the model [18]. By considering random effects and correlations among quadrats, GLMMs effectively manage such dependencies, reducing biases and improving model fit and predictive accuracy [58].

To optimize and simplify our GLMMs, we utilized the “MuMin” package version 1.47.5 [59] to generate all candidate models via the “dredge” function and ranked them based on the small-sample-size corrected Akaike Information Criterion (AICc). The most effective or dominant model was identified as the one with the lowest AICc value [60]. We ultimately selected models with ΔAICc < 1 and determined the best model using Akaike weights (AICc wi).

To evaluate the contribution of each predictor variable to the overall marginal R^2^, we applied the “glmm.hp” package version 0.1-2 [61] to perform commonality analysis based on the average shared variance method. This analysis calculated each predictor variable’s unique contribution, represented by the sum of its unique effect and shared variance. The advantage of this approach is its ability to partition the overall marginal R^2^ into unordered contributions for each predictor variable, which automatically sum to the total marginal R^2^ [61]. This method enables a more precise interpretation of the relative importance of each fixed predictor variable in the model.

Shapiro–Wilk tests were performed to assess the normality of ecological factors for the sample plots selected by forest musk deer and roe deer. For normally distributed factors, t-tests were used for comparisons; non-parametric Mann–Whitney U tests were employed for non-normally distributed factors to explore differences in habitat factors between the sample plots of forest musk deer and roe deer. ANOVA was conducted to analyze differences for unordered categorical variables such as vegetation type. A *p*-value of <0.05 was considered statistically significant for differences in habitat variables between the two species.

## 3. Results

### 3.1. Species Identification and Number of Valid Sample Plots

This study collected 299 fecal samples from ungulates, resulting in 227 sample plots. DNA was successfully extracted from all fecal samples, identifying 163 as forest musk deer feces and 136 as roe deer feces. A total of 100 sample plots were utilized by forest musk deer, 71 by roe deer, 12 as coexistence plots, and 44 as control plots. Furthermore, forest musk deer had 112 utilized plots compared to 115 control plots, while roe deer had 83 utilized plots against 144 control plots. Notably, fecal size and appearance did not effectively distinguish between forest musk deer and roe deer feces.

### 3.2. Model Analysis of Defecation Sites for Forest Musk Deer and Roe Deer

The “Faraway” package version 1.0.8 calculations indicated no variables with VIF values greater than 5 for either species. Therefore, all variables were included in the GLMM model analysis.

The top seven candidate models for forest musk deer with ΔAICc < 1 included slope, aspect, elevation, tree diversity, average tree height, shrub height, herbaceous cover, and herb height, totaling eight environmental variables (Table 2). The models revealed that slope and tree diversity significantly impacted the defecation site preferences of forest musk deer (*p* < 0.01). In contrast, elevation and average herbaceous cover had significant effects (*p* < 0.05). The contribution percentages for the parameters were average herb height (33.73%), average herbaceous cover (19.93%), slope (15.79%), tree diversity (15.64%), and elevation (4.26%). Parameter estimates from the GLMM model indicated that forest musk deer on Huanglong Mountain preferred defecation sites in the mid-to-lower mountain regions with lower herbaceous cover and height, and greater tree diversity and slope (Table 3).

The top five candidate models for Siberian roe deer with ΔAICc < 1 included six environmental variables: slope position, elevation, average tree height, shrub diversity, covertness, and average herbaceous cover (Table 4). The models indicated that elevation significantly influenced the defecation site preferences of Siberian roe deer (*p* < 0.001). Additionally, average herbaceous cover also showed a significant effect (*p* < 0.01) (Table 5). These two factors were the most important in the model, accounting for 54.63% and 29.31% of the variance, respectively. Moreover, slope position contributed 10.24% to the model. Thus, parameter estimates from the GLMM model indicated that Siberian roe deer on Huanglong Mountain prefer defecation sites in the mid-to-upper mountain regions with low herbaceous cover.

### 3.3. Spatial Utilization Differences Between Forest Musk Deer and Siberian Roe Deer

**Figure 2:**
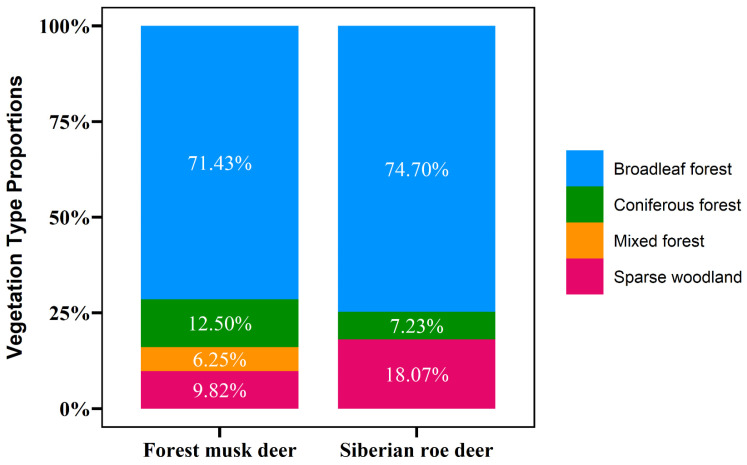
Defecation site vegetation type proportions for forest musk deer and Siberian roe deer.

Analysis of variance (ANOVA) revealed a significant difference in vegetation type utilization between forest musk deer and Siberian roe deer on Huanglong Mountain (*p* = 0.01). The broadleaf forest was the primary habitat for both species, comprising 71.43% of the forest musk deer habitat and 74.7% of the roe deer habitat (Figure 2). Compared to roe deer, forest musk deer showed a higher utilization rate of coniferous forests (12.5%) and lower utilization of woodland shrub areas (9.85%). Furthermore, roe deer appeared more frequently in shrubland (18.07%) and were absent from mixed conifer–broadleaf forests. Forest musk deer were also found in these mixed forests (6.25%).

**Figure 3:**
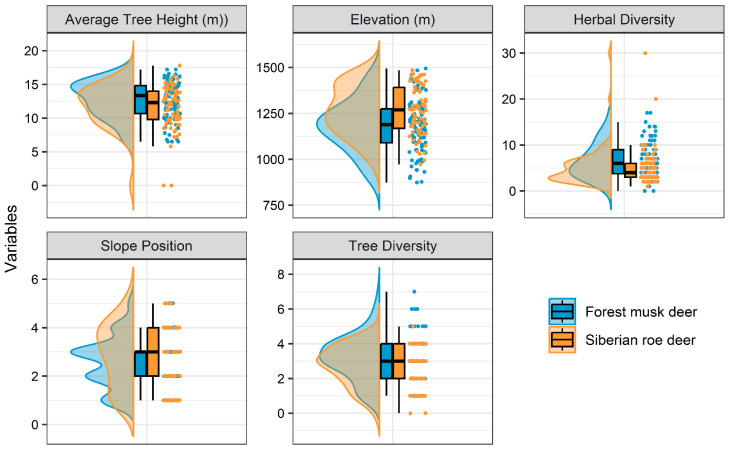
Comparison of environmental variables showing significant differences in defecation sites between forest musk deer and Siberian roe deer.

According to the Shapiro–Wilk test, all datasets except tree numbers did not meet normality assumptions. Therefore, Mann–Whitney U tests were applied to all non-normally distributed data (Table 6). Results indicated significant differences between the two species in elevation (*p* < 0.001) and herbaceous diversity (*p* < 0.01). Notable differences were observed in slope position, tree diversity, and average tree height (*p* < 0.05); however, these differences, while statistically significant, were relatively minor and may have limited ecological significance. Compared to roe deer, forest musk deer defecation sites were located at lower elevations (1179.99 m ± 141.24 m), on lower slope positions (2.53 ± 1.04), and featured greater tree diversity (3.18 ± 1.28), higher average tree height (12.77 m ± 2.73 m), and increased herbal diversity (6.51 ± 3.82). Thus, elevation, slope position, tree diversity, average tree height, and herbal diversity are key factors defining spatial utilization differences between forest musk deer and Siberian roe deer (Figure 3).

## 4. Discussion

Mammal preference for defecation sites is a spatially attributed behavior [16,62] and reflects microhabitat selection. By integrating environmental factor surveys, we can examine the spatial utilization of specific animals and the ecological characteristics of sympatric species at the same trophic level [63]. This study utilized defecation site data to investigate the microhabitat preferences of forest musk deer and Siberian roe deer, effectively capturing habitat use at a fine spatial scale. For elusive species like the forest musk deer, defecation site analysis minimizes disturbance and provides an efficient method to infer habitat preferences. Integrating this data with environmental variables enabled a detailed analysis of spatial ecological segregation.

However, this approach has limitations. Defecation site data alone may not fully reflect habitat use, overlooking seasonal variations and non-defecation behaviors. Additionally, the reliance on infrared camera data for site selection could introduce sampling bias by excluding unmonitored areas. Future studies should incorporate direct observations and broader spatial sampling to complement and validate these findings.

### 4.1. The Impact of Abiotic Factors on the Defecation Site Preferences of Forest Musk Deer and Siberian Roe Deer

In this study, we examined three abiotic factors—elevation, slope position, and slope—concerning the defecation site preferences of forest musk deer and Siberian roe deer. Results indicated that slope and elevation significantly influenced forest musk deer defecation sites. GLMM analysis also revealed that forest musk deer preferred steeper terrains (Table 3), aligning with previous studies [18,25,64]. This preference is likely due to the well-developed hind limbs of musk deer, enabling them to escape quickly from predators in steep areas [65]. Research by Hu [66] and Yang [67] confirmed that forest musk deer tend to favor steep regions while avoiding flat slopes of 0°–5°. Moreover, some studies suggest that forest musk deer prefer high-altitude habitats, attributing this to cooler conditions or fewer human disturbances at higher elevations [27,64,68]. In contrast to these results, this study found that forest musk deer on Huanglong Mountain selected mid-to-lower mountain areas. Therefore, we propose that the steep terrain in these lower regions, along with the relatively flat mid-upper areas and minimal human disturbance, contributes to the preference for these lower elevations. Thus, in the absence of human disturbance, slope is a key factor influencing the selection of relatively high or low elevations by forest musk deer.

For Siberian roe deer, defecation sites were more frequently found at higher elevations (Table 5), with elevation contributing over half (54.63%) to their preference model. Previous research indicates that the probability of roe deer presence increases with elevation in the Greater Khingan Mountains [69]. In contrast, in the Huangni River Nature Reserve, roe deer tend to favor lower elevations. Our findings indicate that Siberian roe deer prefer mid-to-upper mountain regions with relatively gentle terrain, consistent with research by Qi [70]. We hypothesize that, similar to the ecological and biological reasons for forest musk deer, the body structure of roe deer makes them less suited to steep terrains, leading them to favor more gentle environments. Thus, an interspersed climate of relatively steep and gentle terrains may facilitate the coexistence of both species.

### 4.2. Influence of Biotic Factors on Forest Musk Deer and Siberian Roe Deer Defecation Site Preferences

Defecation site preferences of forest musk deer and Siberian roe deer were assessed regarding 13 biotic factors across three vegetation layers: tree layer (canopy cover, tree diversity, tree density, average tree height, average diameter at breast height (DBH), and the number of trees with DBH > 30 cm), shrub layer (shrub diversity, shrub density, shrub cover, and average shrub height), and herbaceous layer (herbaceous diversity, herbaceous cover, and average herb height). Notably, these factors provide both food resources and essential concealment. As such, the GLMM results indicated differing influences of these biotic factors on defecation site preferences for the two species.

Our study found that tree diversity significantly impacted the defecation site preference for forest musk deer. In contrast, no significant effect of tree layer factors was observed for roe deer. Prior research suggests that roe deer rarely feed on tree leaves [71,72], while forest musk deer consume tree leaves more frequently [73]. Additionally, the presence of tree branches provides musk deer with opportunities to escape predators by leaping [65]. Thus, high tree diversity implies good canopy cover and concealment. It may also offer critical escape routes for forest musk deer, an advantage not applicable to roe deer.

While previous dietary studies show that young shrub leaves are essential in the diets of musk deer and roe deer [72,74,75,76] and can also provide cover [18,77], this study found no significant preference for shrub layer factors for either species. Moreover, forest musk deer negatively correlated with shrub height (Table 3). We suggest that in the Huanglong Mountain region, the well-developed secondary shrub forests provide abundant food resources. Moreover, the main differences in the shrub layer relate to height, which impacts browsing accessibility for smaller forest musk deer but less for Siberian roe deer.

Both forest musk deer and Siberian roe deer preferred defecation sites with low herbaceous cover (Table 3 and Table 5). GLMM results also indicated that herbaceous cover and height accounted for 53.66% of the model’s explanatory power for musk deer, while herbaceous cover alone accounted for 29.31% in the roe deer model. Thus, we infer that a well-developed tree and shrub layer, which limits sunlight, reduces herbaceous cover, indirectly indicating a selection for environments with adequate tree and shrub cover. Additionally, Sheng [65] noted that musk deer, while requiring forest environments, seldom rest in dense shrubs or tall grass due to limited visibility and escape potential. Similarly, Tian [69] and Qi [70] found that roe deer prefer open areas, facilitating movement and escape.

### 4.3. Spatial Ecological Segregation Between Forest Musk Deer and Siberian Roe Deer

The defecation site preferences of forest musk deer and Siberian roe deer in Huanglong Mountain revealed highly significant differences in elevation and herbaceous diversity (*p* < 0.01), indicating spatial ecological segregation in microhabitat selection. In addition, there were statistically significant differences in tree diversity, average tree height, and slope position (*p* < 0.05). However, these differences were relatively minor compared to those in elevation and herbaceous diversity, suggesting that their contribution to the microhabitat segregation may be limited. These slight differences could reflect variations in habitat structure preferences rather than being the primary drivers of spatial separation.

Among the four vegetation types in the Huanglong Mountain region, forest musk deer primarily utilized broadleaf forests, coniferous forests, mixed conifer–broadleaf forests, and sparse shrub woodlands. In comparison, roe deer mainly used broadleaf forests, sparse shrub woodlands, and coniferous forests, showing a significant difference (*p* = 0.01) (Figure 2). Notably, both species extensively overlapped in broadleaf forest utilization, with 71.43% of forest musk deer and 74.7% of roe deer found in this habitat type, suggesting limited spatial ecological segregation at the habitat scale. This overlap may be attributed to the relatively low elevation gradient in the Huanglong Mountain region, where the terrain features narrow valleys, steep mid-lower mountain slopes, and gentler upper slopes. The vertical vegetation characteristics are not distinct, and the vegetation types exhibit a mosaic distribution. Furthermore, forest musk deer prefers mid-lower slopes with steeper gradients in broadleaf forest habitats, while the roe deer prefer mid-upper slopes with gentler gradients in broadleaf forest habitats (Table 6, Figure 3), indicating clear spatial ecological segregation.

## 5. Conclusions

This study demonstrates that the defecation site preferences of forest musk deer and Siberian roe deer in the Huanglong Mountain region are influenced by differences in terrain slope and tree diversity, with predator avoidance and escape potential likely being primary drivers. Notably, these differences in defecation site preferences contribute to the distinct utilization of vegetation types across slope positions. As such, it results in spatial ecological segregation at both microhabitat and habitat scales.

## Figures and Tables

**Table 1:** Habitat variables recorded in sample plots.

Habitat Variable	Data Description
Elevation (m) (E)	The elevation of the center of the 10 × 10 m plot
Slope (°) (S)	The slope of the 10 × 10 m plot
Aspect (A)	The aspect of the 10 × 10 m plot
Slope Position (SP)	The slope position of the 10 × 10 m plot
Covertness (m) (C)	A 1-m pole was placed at the quadrat center. Researchers walked in four cardinal directions until the pole was no longer visible, measured the distances, and calculated the average.
Tree Coverage (T_C)	The percentage of ground covered by the upper canopy of vegetation in the 10 × 10 m plot
Tree Diversity (T_DI)	The number of tree species in the 10 × 10 m plot
Tree Density (T_DE)	The total number of trees in the 10 × 10 m plot
Average Tree Height (m) (ATH)	The average height of trees in the 10 × 10 m plot
Average diameter at breast height of trees (cm) (DBH)	The average diameter at breast height (DBH) of trees in the 10 × 10 m plot
Diameter at Breast Height: greater than 30 cm (Dbhg30)	The number of trees with a DBH greater than 30 cm in the 10 × 10 m plot
Shrub Diversity (S_DI)	The number of shrub species in the 5 × 5 m plot
Shrub Density (S_DE)	The total number of shrubs in the 5 × 5 m plot
Shrub Canopy (S_C)	The percentage of ground area covered by the projection of shrub canopies in the 5 × 5 m plot
Average Shrub Height (m) (ASH)	The average height of shrubs in the 5 × 5 m plot
Herbal Diversity (HD)	The number of herbaceous species across four 1 × 1 m plots
Average Herbaceous Cover (AHC)	The average cover of herbaceous plants across four 1 × 1 m plots
Average Herb Height (m) (AHH)	The average height of herbaceous plants across four 1 × 1 m plots

**Table 2:** Selected candidate models explaining forest musk deer latrine site selection patterns.

Models	Variables	df	logLik	AICc	Delta	Weight
1	T_DI + S + E + ASH + AHC	7	−122.5114	259.5342	0.0000	0.0579
2	T_DI + ATH + S + E + ASH +AHC	8	−121.5188	259.6981	0.1639	0.0533
3	T_DI + ATH + S+ E + ASH + AHC+ AHH	9	−120.5267	259.8829	0.3487	0.0486
4	T_DI + S + E + ASH + AHC + AHH	8	−121.6952	260.0510	0.5168	0.0447
5	T_DI + A+ S + E + ASH + AHC	8	−121.7049	260.0704	0.5363	0.0443
6	T_DI + S+ E + AHC	6	−123.8530	260.0879	0.5537	0.0439
7	T_DI + A + S + E + ASH + AHC + AHH	9	−120.7451	260.3196	0.7855	0.0391

S: Slope; A: Aspect; E: Elevation; T_DI: Tree Diversity; ATH: Average Tree Height; ASH: Average Shrub Height; AHC: Average Herbaceous Cover; AHH: Average Herb Height.

**Table 3:** Parameter estimates (estimate) and relative importance (I.perc) of environmental variables in the top generalized linear mixed models (GLMMs) for the selection of latrine sites for forest musk deer.

Variables	Estimate	Std. Error	Z Value	Pr (>|z|)	I.perc (%)
(Intercept)	6.575242	2.818556	2.333	0.01966 *	
S	0.048079	0.017765	2.706	0.00680 **	15.79
A	0.674537	0.591218	1.141	0.25390	1.38
E	−0.005199	0.002095	−2.482	0.01308 *	4.26
T_DI	0.529648	0.186624	2.838	0.00454 **	15.64
ATH	−0.088258	0.071005	−1.243	0.21387	3.85
ASH	−0.470024	0.242346	−1.939	0.05244	5.41
AHC	−2.556200	1.148427	−2.226	0.02603 *	19.93
AHH	−4.005994	2.800626	−1.430	0.15260	33.73

S: Slope; A: Aspect; E: Elevation; T_DI: Tree Diversity; ATH: Average Tree Height; ASH: Average Shrub Height; AHC: Average Herbaceous Cover; AHH: Average Herb Height. * *p* < 0.05; ** *p* < 0.01.

**Table 4:** Selected candidate models explaining Siberian roe deer latrine site selection patterns.

Models	Variables	df	logLik	AICc	Delta	Weight
1	E + AHC	4	−120.1152	248.4106	0.0000	0.0944
2	E + AHC + C	5	−119.2310	248.7334	0.3228	0.0804
3	E + S_DI + AHC	5	−119.3063	248.8841	0.4735	0.0745
4	ATH +E + AHC	5	−119.3694	249.0103	0.5997	0.0700
5	SP + E + AHC + C	6	−118.4382	249.2582	0.8476	0.0618

SP: Slope Position; E: Elevation; ATH: Average Tree Height; S_DI: Shrub Diversity; C: Covertness; AHC: Average Herbaceous Cover.

**Table 5:** Parameter estimates (Estimate) and relative importance (I.perc) of environmental variables in the top generalized linear mixed models (GLMMs) for the selection of latrine sites for Siberian roe deer.

Variables	Estimate	Std. Error	Z Value	Pr (>|z|)	I.perc (%)
(Intercept)	−9.530762	2.957765	−3.222	0.001272 **	
SP	−0.313664	0.208598	−1.504	0.132665	10.42
E	0.008789	0.002306	3.812	0.000138 ***	54.63
ATH	−0.051760	0.062103	−0.833	0.404586	1.78
S_DI	0.100891	0.104420	0.966	0.333940	0.62
C	−0.016903	0.019613	−0.862	0.388787	3.24
AHC	−2.806144	0.917925	−3.057	0.002235 **	29.31

SP: Slope Position; E: Elevation; ATH: Average Tree Height; S_DI: Shrub Diversity; C: Covertness; AHC: Average Herbaceous Cover. ** *p* < 0.01; *** *p* < 0.001.

**Table 6:** Environmental variable comparisons influencing defecation site preferences of forest musk deer and Siberian roe deer.

Variables	Mean ± SD			Statistic	*p*
	Total (*n* = 227)	Forest Musk Deer (*n* = 100)	Siberian Roe Deer (*n* = 71)		
T_DE	9.55 ± 4.60	10.38 ± 3.73	9.73 ± 4.41	t = 1.04	0.301
E (m)	1194.09 ± 147.03	1179.99 ± 141.24	1265.38 ± 134.64	Z = −3.63	<0.001 ***
A	0.47 ± 0.34	0.47 ± 0.34	0.45 ± 0.36	Z = −0.06	0.955
S (°)	24.11 ± 13.36	26.46 ± 13.15	27.37 ± 12.05	Z = −0.71	0.476
C (m)	15.72 ± 13.06	13.98 ± 9.41	14.40 ± 9.65	Z = −0.17	0.868
T_DI	2.75 ± 1.35	3.18 ± 1.28	2.69 ± 1.14	Z = −2.33	0.020 *
TC	0.68 ± 0.25	0.73 ± 0.18	0.69 ± 0.23	Z = −0.84	0.403
ATH (m)	11.62 ± 3.97	12.77 ± 2.73	11.68 ± 3.26	Z = −2.31	0.021 *
DBH (cm)	22.70 ± 7.55	24.43 ± 5.37	23.46 ± 6.02	Z = −0.59	0.556
DBHG30	1.68 ± 1.68	1.84 ± 1.78	1.66 ± 1.64	Z = −0.56	0.573
S_DI	5.21 ± 2.31	4.97 ± 1.79	4.97 ± 1.76	Z = −0.18	0.857
S_DE	48.81 ± 47.29	43.73 ± 36.42	46.68 ± 38.93	Z = −0.31	0.760
SC	0.37 ± 0.27	0.34 ± 0.25	0.36 ± 0.23	Z = −0.64	0.520
ASH (m)	2.24 ± 0.84	2.14 ± 0.82	2.31 ± 0.78	Z = −0.74	0.459
HD	7.31 ± 5.41	6.51 ± 3.82	5.17 ± 4.18	Z = −2.84	0.005 **
AHC	0.29 ± 0.31	0.20 ± 0.20	0.17 ± 0.19	Z = −1.47	0.141
AHH (m)	0.18 ± 0.15	0.14 ± 0.07	0.14 ± 0.09	Z = −0.15	0.880
SP	2.47 ± 1.17	2.53 ± 1.04	2.87 ± 1.26	Z = −2.00	0.046 *

* *p* < 0.05; ** *p* < 0.01; *** *p* < 0.001.

## Data Availability

The original contributions presented in this study are included in the article. Further inquiries can be directed to the corresponding authors.
